# Development of a Model for Quantitative Assessment of Newborn Screening in Japan Using the Analytic Hierarchy Process

**DOI:** 10.3390/ijns9030039

**Published:** 2023-07-14

**Authors:** Keiko Konomura, Eri Hoshino, Kotomi Sakai, Takashi Fukuda, Go Tajima

**Affiliations:** 1 Center for Outcomes Research and Economic Evaluation for Health (C2H), National Institute of Public Health, Wako-shi 351-0197, Japan; konomura.k.aa@niph.go.jp (K.K.); fukuda.t.aa@niph.go.jp (T.F.); 2 Division of Policy Evaluation, Department of Health Policy, Research Institute, National Center for Child Health and Development, Tokyo 157-8535, Japan; hoshino-e@ncchd.go.jp; 3 Comprehensive Unit for Health Economic Evidence Review and Decision Support (CHEERS), Research Organization of Science and Technology, Ritsumeikan University, Kyoto 600-8815, Japan; ko-sakai@fc.ritsumei.ac.jp; 4 Division of Neonatal Screening, Research Institute, National Center for Child Health and Development, Tokyo 157-8535, Japan

**Keywords:** newborn screening, inherited disorder, public health, pediatrics, rare diseases, selection criteria

## Abstract

Whether or not conditions should be included in publicly funded newborn screening (NBS) programs should be discussed according to objective and transparent criteria. Certain criteria have been developed for the introduction of NBS programs in the context of individual countries; however, there are no standard selection criteria for NBS programs in Japan. This study aimed to develop a quantitative scoring model to assess newborn screening that incorporates the views of a variety of stakeholders in Japan. The five recommended eligibility criteria for NBS were stratified based on previous studies and expert opinions, using the analytic hierarchy process. We conducted a cross-sectional, web-based questionnaire targeting a wide range of people involved in NBS to investigate pairwise comparisons of the evaluation items between February and April of 2022. There were 143 respondents. Most of our respondents (44.1%) were physicians. Fifty-eight respondents (40.6%) had been engaged in NBS-related research or work for more than 10 years. The distribution of allocation points was the highest for ‘intervention’, ‘screening test’, ‘follow-up setting’, ’economic evaluation’, and ’disease/condition’, in that order. The algorithm in this study will guide decision makers in collecting and evaluating objective data, thus enabling transparent discussions to occur.

## 1. Introduction

Newborn screening (NBS) is a public health program aimed at screening newborns for conditions that can lead to death or severe disability if left undetected [1]. Early detection of such conditions allows for prompt intervention to reduce or avoid the undesirable condition. The implementation of NBS varies widely depending on national government policies, ranging from countries that screen for many conditions to countries that do not implement NBS [2].

The NBS program in Japan started in 1977 as a public health program [3]. The introduction of tandem mass spectrometry in 2013 has increased the number of conditions screened for. As of 2022, the NBS program covers 20 conditions in all municipalities. The targeted conditions are congenital disorders of endocrinology and metabolism for which diet and relatively inexpensive drug therapy are effective. In recent years, the development of innovative treatment methods and improvements in testing techniques have led to discussions on how to further expand the current NBS program. Adding new conditions to the NBS requires careful and transparent discussions on the benefits and harms of a screening program among the relevant stakeholders.

As principles for evaluating conditions for screening, Wilson and Jungner’s principles of screening are still an important perspective today [4]. On this basis, many principles have been developed for the introduction of NBS within the context of each country [5,6,7,8,9]. The Advisory Committee on Heritable Disorders in Newborns and Children (ACHDNC) in the United States has described the process of adding a condition to the Recommended Uniform Screening Panel [8]. The process requires that the candidate condition is nominated according to uniform evaluation criteria and after multiple steps of the review process, the evaluation results are organized by a decision matrix, and a final decision is made. The UK National Screening Committee (UK NSC) has established criteria for objective evaluation based on evidence from screening programs [9]. However, in Japan, there are no standard criteria for selecting conditions for NBS programs. This study aimed to develop a quantitative scoring model to assess newborn screening that incorporates the views of a variety of stakeholders in Japan.

## 2. Materials and Methods

The study was approved by the Institutional Review Board of the National Institute of Public Health, Japan (#706).

### 2.1. Analytic Hierarchy Process

We applied the analytic hierarchy process (AHP) in the development of our scoring algorithm. The AHP, one of the decision support tools [10,11], is used not only in healthcare research but also in many other fields [12,13]. The AHP decomposes complex decision-making issues into a hierarchical structure and calculates quantitative weights via pairwise comparisons of each component of the hierarchical structure. The advantage of pairwise comparisons is that they facilitate comparisons of complex problems and deal with subjective perspectives. We used the AHP in three steps. The first step was to identify the evaluation components of the issue and stratify them into a hierarchical structure. The second step was to perform a pairwise comparison of each component of the hierarchical structure. In the third step, the weights of the evaluation components were calculated based on the values obtained in the second step.

### 2.2. Structuring the Assessment Items

We conducted a literature review to select items to be assessed when considering the addition of a condition to the NBS. Twenty-two clinical and NBS experts discussed these items, after which they selected and tailored the structure of the assessment items to the Japanese context. A pilot study of the pairwise comparisons was then conducted within the study group, and the structure of the assessment items was reconsidered (Table 1). The items were finally classified into five categories: disease/condition, screening test, intervention, follow-up setting, and economic evaluation.

### 2.3. Participants and the Questionnaire

We conducted a cross-sectional, questionnaire-based survey to anonymously investigate pairwise comparisons of the evaluation items between February and April of 2022. The subjects included members of the board of directors of societies related to NBS, employees of facilities that perform NBS, employees of local government departments related to NBS, representatives of patient organizations, medical students, and healthcare workers whose work is not related to NBS (hereinafter referred to as ‘general healthcare workers‘). Invitations to participate in the survey were sent via e-mail, together with the survey’s URL link, except for those to general healthcare workers. Six general healthcare workers were selected from the survey panel provider. The extracted features were as follows: type of job (doctor, pharmacist, or nurse), sex, with/without children. General healthcare workers were invited to take part in individual online interviews so that they could ask questions about NBS expertise.

The questionnaire was prepared on a dedicated website in Japanese. It consisted of a description of the assessment items, a pairwise comparison questionnaire, and an open-ended question about the survey. There were 88 questions (10 in categories, 22 in sub-categories, and 56 in criteria) for pairwise comparisons, and the response options were equal, moderate importance, strong importance, and very strong importance (Table S1). In the pairwise comparison section, participants were presented with two evaluation items for each question and asked to compare the relative importance of the evaluation items in selecting conditions for NBS. If respondents had difficulties with technical content about the NBS, they were allowed to skip the 56 questions in criteria that required the most technical knowledge.

### 2.4. Determination of Allocated Points

Weights were calculated using the geometric mean of the AHP. The consistency index (CI) was used to assess the consistency of responses. The CI for a completely consistent response was 0, and a CI greater than 0.15 was considered inconsistent. The calculated weights were multiplied by 1000 and rounded off to the nearest whole numbers to determine the allocation of points for each evaluation item. Data analysis was performed using SAS 9.4 (SAS Institute Inc., Cary, NC, USA) and Microsoft Excel 2019.

### 2.5. Validation of the Scoring Algorithm

We applied scoring algorithms for phenylketonuria, medium-chain acyl-CoA dehydrogenase (MCAD) deficiency, and congenital hypothyroidism (CH). These diseases are expected to score high because they are regarded as the most suitable diseases for NBS and have already been included in the NBS program in Japan. A literature review was conducted to examine the existing evidence, and disease experts in the study group evaluated this evidence.

## 3. Results

### 3.1. Participants

A total of 156 respondents participated in the study. The analysis included 143 respondents who completely answered the categories and the subcategories (95 respondents answered all questions). Males accounted for 57.3% of the respondents, and many (31.5%) of the respondents were in their 50s (Table 2). Physicians were the most represented professionals among the respondents (44.1%), followed by laboratory technicians (14.0%). Fifty-eight respondents (40.6%) had been engaged in NBS-related research or had been working for more than 10 years.

### 3.2. Calculated Weights and Scores

**Figure 1:**
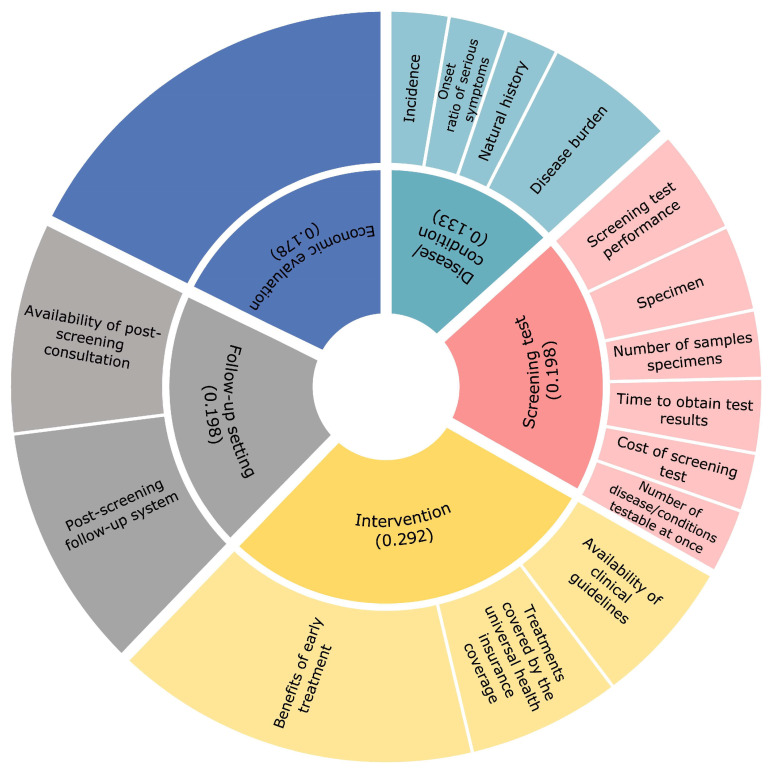
Calculated weight distribution.

The calculated weights were 0.292 for the ‘intervention’, 0.198 for the ‘screening test’, 0.198 for the ‘follow-up setting’, 0.178 for the ‘economic evaluation’, and 0.133 for the ‘disease/condition ‘(Figure 1). CIs were below 0.15 for all items. The final allocation of scores is shown in Table 3. The highest possible score was 620 points, and the lowest possible score was 114 points. The results of the evaluation of the current NBS target diseases, phenylketonuria, MCAD deficiency, and CH were 609, 605, and 540 points, respectively (Table 4, Table 5 and Table 6).

The pie chart shows the weight distribution assigned to each evaluation item as a result of a pairwise comparison by the subject. The larger the weight assigned, the larger the representative area of the figure, which means that it is more important to the respondents. The inner circle indicates the weight distribution assigned to each of the five categories. The numbers indicate the percentage of weight assigned and the total value is 1 (although, due to rounding issues, the numbers in the figure do not add up to 1). The outer circles show the relative weight percentage assigned to the subcategories.

## 4. Discussion

The algorithm developed in this study helps decision makers to gather objective evidence and consider the priorities of target conditions for NBS in Japan. The score assigned to a condition also helps to ensure the transparency of the assessment. We have developed this algorithm primarily focusing on clinical factors. Organizations such as the ACHDNC and UK NSC, which are public bodies involved in evaluating NBS, incorporate elements related to the ethical aspects of the screening program and the feasibility of implementation in public health systems. However, we did not include these elements because we found it challenging to incorporate them into the hierarchical structure. These must be discussed separately from the objective evidence collected using this algorithm, if necessary.

‘Intervention’ was the most highly rated item. In this item, ‘scientific evidence of the benefits of early intervention’ was ascribed the highest score. This item was considered important because the goal of NBS is to improve patient outcomes rather than detect disease early. The fewest points were allocated to ‘disease/condition’. Respondents seemed to consider screening performance and the intervention process for patients more important than a better understanding of conditions. ‘Economic evaluation’ was ranked second from the bottom. Local government employees tended to respond that cost-effectiveness was an important aspect of the NBS.

The three current NBS conditions were evaluated using our algorithm and scored as highly as expected; however, the ‘economic evaluation’ item for CH was deducted 68 points. This is because CH is screened independently during NBS, and has been included in NBS even before more attention was paid to cost-effectiveness; thus, sufficient information was not available for Japanese context. CH is known to have a high incidence and inexpensive treatment can prevent serious clinical consequences. Therefore, screening for CH is explicitly considered to be cost-effective, although no formal analysis has been conducted.

The algorithm does not set a threshold for selecting target conditions because we consider that the threshold should be determined by decision makers according to their context. There is no independent and dedicated organization in Japan that discusses NBS comprehensively and continuously; however, each local municipality makes decisions on its own terms. Therefore, the decision-making process and thresholds were not set as they depend on each municipality’s public health policy. However, if we were to suggest a guide to thresholds, scores obtained for the current NBS-targeted diseases would be helpful.

We cannot rule out the possibility of selection bias for the data used in the development of this algorithm. As the NBS is a public health project, a broadly representative view was required in the development of the algorithm. However, it was difficult to seek in-depth understanding and opinions from the general population because the field of NBS is a highly specialized one. Therefore, we collected the views of individuals from relevant fields of NBS and those with medical backgrounds. Although we encouraged the subjects to join the survey several times, the response rate is considered to be about 30%. Some of the subjects may not have responded to the questions because they thought they did not have the expertise to answer the questions properly or they did not think it was relevant to them. It is also possible that the subjects were busy and may have given up on answering the questions due to the large number of questions. Because 66% of the respondents have NBS-related work or research experience, the score reflects more input from subjects who are more interested in NBS.

## 5. Conclusions

The number of candidate conditions for NBS is increasing due to recent developments in genomic technologies and improvements in screening techniques. The selection of such NBS candidate conditions in Japan requires multi-criterion decision making. The algorithm in this study will guide decision makers to collect and evaluate objective data, which will facilitate transparent discussions. It is also expected to provide a reference for data to be investigated by those who wish to expand the NBS. Future revisions will be necessary because it is possible that further advances in medical care and social changes will lead to changes in the assessment items and the distribution of scores.

## Figures and Tables

**Table 1:** Structure of the assessment items.

Categories	Subcategories (Criteria)	
Disease/condition	1.	Incidence of the disease/condition
	(≥1/20,000, ≥1/50,000 but <1/20,000, ≥1/100,000 but <1/50,000, ≥1/200,000 but <1/100,000, <1/200,000)	
	The incidence of the disease/condition should be adequately understood.	
	2.	Onset of serious symptoms within 96 h of birth *
	(No onset, ≥1% but <30%, ≥30% but <70%, ≥70% or unknown)	
	The incidence of serious symptoms observed before obtaining screening test results should be clear in order to obtain optimal outcomes for early detection and treatment via screening.	
	3.	Natural history of the disease/condition
	(clear; unclear)	
	The natural history of the disease/condition and their variant forms concerned should be adequately understood.	
	4.	Disease burden without treatment
	(high disease burden; moderate disease burden; low disease burden)	
	The disease burden of the untreated disease/condition and its variant forms should be adequately understood and it should be a significant health problem.	
Screening test	5.	Screening test performance
	(high sensitivity and specificity; high sensitivity but low specificity; others)	
	Screening test performance should be adequately precise and validated.	
	6.	Availability of dried blood specimens
	(Yes, No)	
	Collecting samples as dried blood specimens is the principal approach. In the case of other methods of specimen collection, it should be simple and less invasive.	
	7.	Number of samples that can be processed
	(≥200 samples/day/full-time equivalent (FTE), ≥100 but <200 samples/day/FTE, <100 samples/day/FTE)	
	The facility where newborn screening is performed should be able to process a sufficient volume of specimens.	
	8.	Time to obtain screening test results
	(<1 day, ≥1 but <2 days, ≥2 days)	
	The time taken to obtain screening test results should be clear.	
	9.	Cost of screening test (Japanese yen, JPY)
	(<500 JPY, 500–999 JPY, 1000–4999 JPY, ≥5000 JPY)	
	The additional costs of introducing the screening test should be clear.	
	10.	Number of diseases/conditions testable at once
	(≥4, 2–3, 1)	
	Screening tests can measure multiple items simultaneously and should efficiently detect multiple diseases.	
Intervention	11.	Availability of clinical guidelines
	(available; partially available; not available)	
	There should be established, evidence-based, agreed clinical guidelines covering cut-off points for testing, additional testing, and diagnosis for subjects with positive screening tests, policies for individuals to whom interventions should be provided, and standard and effective treatment strategies.	
	12.	Availability of medical interventions covered by national health insurance
	(available; partially available; other)	
	NBS participants with positive screening tests should receive appropriate interventions within the national health insurance system.	
	13.	Scientific evidence of the benefits of early intervention
	(yes; some; no)	
	There should be scientific evidence that patients identified via screening tests can benefit from appropriate early intervention.	
Follow-up setting	14.	Post-screening follow-up system
	(well established; partially established; not established)	
	After the patient has been diagnosed, there should be a core hospital that has a specialist on the disease/condition within the accessible range. A system to coordinate cooperation between the local hospital that the patient visits routinely or on an emergency basis and the hospital with a specialist should be well established.	
	15.	Availability of post-screening consultation
	(available; partially available; not available)	
	A system that can sufficiently explain the disease to the patients and family members who tested positive and the patient’s family (e.g., genetic counseling, brochures to explain the disease, and contacts for inquiries) should be well established. Furthermore, this system should be standardized nationwide to allow providing information fairly to all individuals identified through screening.	
Economic evaluation	16.	Economic evaluation
	(scientific evidence is available; some scientific evidence is available; other)	
	An economic evaluation of the screening program should include appropriate resources used and health outcomes simultaneously reflecting the national context.	

The structure of the evaluation items to add conditions to the NBS is divided into three levels: ‘Categories’ are the largest classification, describing the five representative aspects of the evaluation items; ‘Subcategories’ are intermediate classifications, describing specific evaluation items; and ‘Criteria’ are classifications that define the criteria for the subcategories. * Dried blood specimens for NBS are usually collected on the 4th day after birth.

**Table 2:** Characteristics of the included respondents (*n* = 143).

Characteristic		*n*	(%)
Age	20–29	18	(12.6)
	30–39	19	(13.3)
	40–49	25	(17.5)
	50–59	45	(31.5)
	60–69	27	(18.9)
	70–79	3	(2.1)
	Over 80 years old	2	(1.4)
	Missing	4	(2.8)
Sex	Male	82	(57.3)
	Female	58	(40.6)
	Missing	3	(2.1)
With/without children	No	46	(32.2)
	Yes	92	(64.3)
	Missing	5	(3.5)
Occupations or organization	Physician	63	(44.1)
	Clinical laboratory technician	20	(14.0)
	Medical student	17	(11.9)
	Patient advocacy group	12	(8.4)
	Local government employees	10	(7.0)
	Nurse	6	(4.2)
	NBS-related inspection technician	5	(3.5)
	Midwife	4	(2.8)
	Pharmacist	3	(2.1)
	Others	1	(0.7)
	Missing	2	(1.4)
Clinical department	Pediatrics	50	(35.0)
	Obstetrics and Gynecology	4	(2.8)
	Pediatric Surgery	3	(2.1)
	Neonatology	3	(2.1)
	Others	5	(3.5)
	Missing	78	(54.5)
Academic affiliations (multiple choices allowed)	The Japanese Society for Neonatal Screening	54	(37.8)
	The Japan Society of Human Genetics	39	(27.3)
	The Japanese Society for Pediatric Gastroenterology, Hepatology, and Nutrition	24	(16.8)
	The Japanese Society for Inherited Metabolic Diseases	21	(14.7)
	The Japan Society of Perinatal and Neonatal Medicine	20	(14.0)
	The Japanese Society for Pediatric Endocrinology	17	(11.9)
	The Japanese Society for Genetic Counseling	16	(11.2)
	The Japanese Society of Child Neurology	14	(9.8)
	The Japan Society for Neonatal Health and Development	12	(8.4)
	The Japanese Society for Pediatric Infectious Diseases	10	(7.0)
	The Japan Society of Obstetrics and Gynecology	7	(4.9)
	The Japan Association of Obstetricians and Gynecologists	5	(3.5)
	The Japanese Society for Immunodeficiency and Autoinflammatory Diseases	5	(3.5)
	The Japan Academy of Midwifery	2	(1.4)
	The Japanese Midwives Association	2	(1.4)
	The Japanese Society of Genetic Nursing	2	(1.4)
Years of experience in NBS-related work or research	More than 10 years	58	(40.6)
	None	32	(22.4)
	1–4 years	26	(18.2)
	5–9 years	11	(7.7)
	Missing	16	(11.2)

**Table 3:** Allocation of scores.

Categories	Subcategories	Criteria	Score
Disease/condition	Incidence of the disease/condition	≥1/20,000	10
		≥1/50,000 but <1/20,000	7
		≥1/100,000 but <1/50,000	5
		≥1/200,000 but <1/100,000	3
		<1/200,000	2
	Onset of serious symptoms within 96 h of birth	No onset	9
		≥1% but <30%	7
		≥30% but <70%	5
		≥70% or unknown	3
	Natural history of the disease/condition	Clear	18
		Unclear	5
	Disease burden without treatment	High disease burden	37
		Moderate disease burden	15
		Low disease burden	6
Screening test	Screening test performance	High sensitivity and specificity	32
		High sensitivity but low specificity	10
		The others	5
	Availability of dried blood specimens	Yes	29
		No	8
	Number of samples that can be processed	≥200 samples/day/full-time equivalent (FTE)	16
		≥100 but <200 samples/day/FTE	9
		The others	4
	Time to obtain screening test results	<1 day	17
		≥1 but <2 days	11
		≥2 days	5
	Cost of screening test	<500 Japanese yen (JPY)	12
		500–999 JPY	8
		1000–4999 JPY	4
		≥5000 JPY	2
	Number of diseases/conditions testable at once	≥4	16
		2–3	8
		1	4
Intervention	Availability of clinical guidelines	Available	39
		Partially available	19
		Not available	8
	Availability of medical interventions covered by national health insurance	Available	41
		Partially available	17
		The others	8
	Scientific evidence for the benefits of early intervention	Yes	104
		Some	41
		No	16
Follow-up setting	Post-screening follow-up system	Well-established	66
		Partially established	29
		Not established	11
	Availability of post-screening consultation	Available	60
		Partially available	23
		Not available	9
Economic evaluation	None	Scientific evidence is available	114
		Some scientific evidence is available	46
		The others	18

This table shows the final distribution of the scores assigned as a result of the survey. Higher scores indicate items that were rated more important by the respondents. Criteria in the subcategories are sorted in descending order of score. The maximum score is 620 and the minimum score is 114 in this scoring model.

**Table 4:** Scoring results of phenylketonuria.

Disease/Condition				
Categories	Subcategories	Criteria	Rating Score	Full Marks
Disease/condition	Incidence of the disease/condition	≥1/50,000 but <1/20,000	7	10
	Onset of serious symptoms within 96 h of birth	No onset	9	9
	Natural history of the disease/condition	Clear	18	18
	Disease burden without treatment	High disease burden	37	37
Subtotal			71	74
Screening test	Screening test performance	High sensitivity and specificity	32	32
	Availability of dried blood specimens	Yes	29	29
	Number of samples that can be processed	≥200 samples/day/full-time equivalent (FTE)	16	16
	Time to obtain screening test results	<1 day	17	17
	Cost of screening test	1000–4999 JPY	4	12
	Number of diseases/conditions testable at once	≥4	16	16
Subtotal			114	122
Intervention	Availability of clinical guidelines	Available	39	39
	Availability of medical interventions covered by national health insurance	Available	41	41
	Scientific evidence for the benefits of early intervention	Yes	104	104
Subtotal			184	184
Follow-up setting	Post-screening follow-up system	Well established	66	66
	Availability of post-screening consultation	Available	60	60
Subtotal			126	126
Economic evaluation		Scientific evidence is available	114	114
Total score			609	620

The results of applying the developed scoring model to phenylketonuria (PKU) are shown. The rating score represents the score assigned to the selected ‘Criteria’ for the PKU. Deviations from the full marks indicate scores that PKU did not obtain.

**Table 5:** Scoring results for medium-chain acyl CoA dehydrogenase deficiency.

Disease/Condition				
Categories	Subcategories	Criteria	Rating Score	Full Marks
Disease/condition	Incidence of the disease/condition	≥1/100,000 but <1/50,000	5	10
	Onset of serious symptoms within 96 h of birth	≥1% but <30%	7	9
	Natural history of the disease/condition	Clear	18	18
	Disease burden without treatment	High disease burden	37	37
Subtotal			67	74
Screening test	Screening test performance	High sensitivity and specificity	32	32
	Availability of dried blood specimens	Yes	29	29
	Number of samples that can be processed	≥200 samples/day/full-time equivalent (FTE)	16	16
	Time to obtain screening test results	<1 day	17	17
	Cost of screening test	1000–4999 JPY	4	12
	Number of diseases/conditions testable at once	≥4	16	16
Subtotal			114	122
Intervention	Availability of clinical guidelines	Available	39	39
	Availability of medical interventions covered by national health insurance	Available	41	41
	Scientific evidence for the benefits of early intervention	Yes	104	104
Subtotal			184	184
Follow-up setting	Post-screening follow-up system	Well established	66	66
	Availability of post-screening consultation	Available	60	60
Subtotal			126	126
Economic evaluation		Scientific evidence is available	114	114
Total score			605	620

The results of applying the developed scoring model to medium-chain acyl CoA dehydrogenase (MCAD) deficiency are shown. The rating score represents the score assigned to the selected criteria for the MCAD deficiency. Deviations from the full marks indicate scores that MCAD deficiency did not obtain.

**Table 6:** Scoring results for congenital hypothyroidism.

Disease/Condition				
Categories	Subcategories	Criteria	Rating Score	Full Marks
Disease/condition	Incidence of the disease/condition	≥1/20,000	10	10
	Onset of serious symptoms within 96 h of birth	No onset	9	9
	Natural history of the disease/condition	Clear	18	18
	Disease burden without treatment	High disease burden	37	37
Subtotal			74	74
Screening test	Screening test performance	High sensitivity and specificity	32	32
	Availability of dried blood specimens	Yes	29	29
	Number of samples that can be processed	≥200 samples/day/full-time equivalent (FTE)	16	16
	Time to obtain screening test results	<1 day	17	17
	Cost of screening test	<500 Japanese yen (JPY)	12	12
	Number of diseases/conditions testable at once	1	4	16
Subtotal			110	122
Intervention	Availability of clinical guidelines	Available	39	39
	Availability of medical interventions covered by national health insurance	Available	41	41
	Scientific evidence for the benefits of early intervention	Yes	104	104
Subtotal			184	184
Follow-up setting	Post-screening follow-up system	Well established	66	66
	Availability of post-screening consultation	Available	60	60
Subtotal			126	126
Economic evaluation		Some scientific evidence is available	46	114
Total score			540	620

The results of applying the developed scoring model to congenital hypothyroidism (CH) are shown. The rating score represents the score assigned to the selected criteria for CH. Deviations from the full marks indicate scores that CH did not obtain.

## Data Availability

Not applicable.

## Supplementary Materials

The following supporting information can be downloaded at: https://www.mdpi.com/article/10.3390/ijns9030039/s1, Table S1: Pairwise comparisons in the questionnaire.
