# Supplementary material for: Development of a Model for Quantitative Assessment of Newborn Screening in Japan Using the Analytic Hierarchy Process
Source: Int J Neonatal Screen. 2023 Jul 14;9(3):39. doi: 10.3390/ijns9030039 (PMC10366826; doi:10.3390/ijns9030039)
Supplement: Supplementary file 1 [file IJNS-09-00039-s001.zip › IJNS-2427975-supplementary.docx]

Table S1: Pairwise comparisons in the questionnaire

1. Evaluation of categories

**List of five categories**

| 1. | Disease/condition: The condition should be an important health problem and its natural history and incidence should be well known. |
| --- | --- |
| 2. | Screening test: The screening test method should be established. It is important to have a test with sufficiently high sensitivity and specificity that can be integrated into the current newborn screening system, ensuring high efficiency and low cost per analysis. |
| 3. | Intervention: It is important that the condition has clear evidence for early treatment, well-developed practice guidelines, and can be treated within insurance coverage. |
| 4. | Follow-up setting: It is important that expert consultation is available and there is a system that provides adequate explanations to family members who test positive or to the patient’s family. |
| 5. | Economic evaluation: There must be a balance between the cost and effectiveness of treatment. |

|  | (A) is of very strong importance | (A) is of strong importance | (A) is of moderate importance | Equal importance | (B) is of moderate importance | (B) is of strong importance | (B) is of very strong importance |  |
| --- | --- | --- | --- | --- | --- | --- | --- | --- |
| (A) | 〇 | 〇 | 〇 | 〇 | 〇 | 〇 | 〇 | (B) |
| Disease/condition |  |  |  |  |  |  |  | Screening test |
| (A) | 〇 | 〇 | 〇 | 〇 | 〇 | 〇 | 〇 | (B) |
| Disease/condition |  |  |  |  |  |  |  | Intervention |
| (A) | 〇 | 〇 | 〇 | 〇 | 〇 | 〇 | 〇 | (B) |
| Disease/condition |  |  |  |  |  |  |  | Follow-up setting |
| (A) | 〇 | 〇 | 〇 | 〇 | 〇 | 〇 | 〇 | (B) |
| Disease/condition |  |  |  |  |  |  |  | Economic evaluation |
| (A) | 〇 | 〇 | 〇 | 〇 | 〇 | 〇 | 〇 | (B) |
| Screening test |  |  |  |  |  |  |  | Intervention |
| (A) | 〇 | 〇 | 〇 | 〇 | 〇 | 〇 | 〇 | (B) |
| Screening test |  |  |  |  |  |  |  | Follow-up setting |
| (A) | 〇 | 〇 | 〇 | 〇 | 〇 | 〇 | 〇 | (B) |
| Screening test |  |  |  |  |  |  |  | Economic evaluation |
| (A) | 〇 | 〇 | 〇 | 〇 | 〇 | 〇 | 〇 | (B) |
| Intervention |  |  |  |  |  |  |  | Follow-up setting |
| (A) | 〇 | 〇 | 〇 | 〇 | 〇 | 〇 | 〇 | (B) |
| Intervention |  |  |  |  |  |  |  | Economic evaluation |
| (B) | 〇 | 〇 | 〇 | 〇 | 〇 | 〇 | 〇 | (B) |
| Follow-up setting |  |  |  |  |  |  |  | Economic evaluation |

1. Evaluation of sub-categories

**List of Disease/condition items**

| 1. | Incidence of the disease/condition: The incidence of the disease/condition should be adequately understood. |
| --- | --- |
| 2. | Onset of serious symptoms within 96 hours of birth: The incidence of serious symptoms observed before obtaining screening test results should be clear in order to obtain optimal outcomes for early detection and treatment by screening. |
| 3. | Natural history of the disease/condition: The natural history of the disease/condition and their variant forms concerned should be adequately understood. |
| 4. | Disease burden without treatment: The disease burden of the untreated disease/condition and their variant forms should be adequately understood and they should be an important health problem. |

|  | (A) is of very strong importance | (A) is of strong importance | (A) is of moderate importance | Equal importance | (B) is of moderate importance | (B) is of strong importance | (B) is of very strong importance |  |
| --- | --- | --- | --- | --- | --- | --- | --- | --- |
| (A) | 〇 | 〇 | 〇 | 〇 | 〇 | 〇 | 〇 | (B) |
| Incidence of the disease/condition |  |  |  |  |  |  |  | Onset of serious symptoms within 96 hours of birth |
| (A) | 〇 | 〇 | 〇 | 〇 | 〇 | 〇 | 〇 | (B) |
| Incidence of the disease/condition |  |  |  |  |  |  |  | Natural history of the disease/condition |
| (A) | 〇 | 〇 | 〇 | 〇 | 〇 | 〇 | 〇 | (B) |
| Incidence of the disease/condition |  |  |  |  |  |  |  | Disease burden without treatment |
| (A) | 〇 | 〇 | 〇 | 〇 | 〇 | 〇 | 〇 | (B) |
| Onset of serious symptoms within 96 hours of birth |  |  |  |  |  |  |  | Natural history of the disease/condition |
| (A) | 〇 | 〇 | 〇 | 〇 | 〇 | 〇 | 〇 | (B) |
| Onset of serious symptoms within 96 hours of birth |  |  |  |  |  |  |  | Disease burden without treatment |
| (A) | 〇 | 〇 | 〇 | 〇 | 〇 | 〇 | 〇 | (B) |
| Natural history of the disease/condition |  |  |  |  |  |  |  | Disease burden without treatment |

**List of** **Screening test items**

| 1. | Screening test performance: Screening test performance should be adequately precise and validated. |
| --- | --- |
| 2. | Availability of dried blood specimens: Collecting samples as dried blood specimens is the principal approach. In the case of other methods of specimen collection, it should be simple and less invasive. |
| 3. | Number of samples that can be processed: The facility where newborn screening is performed should be able to process a sufficient volume of specimens. |
| 4. | Time to obtain screening test results: The time taken to obtain screening test results should be clear. |
| 5. | Cost of screening test: The additional costs of introducing the screening test should be clear. |
| 6. | Number of disease/conditions testable at once: Screening tests can measure multiple items simultaneously and should efficiently detect multiple diseases. |

|  | (A) is of very strong importance | (A) is of strong importance | (A) is of moderate importance | Equal importance | (B) is of moderate importance | (B) is of strong importance | (B) is of very strong importance |  |
| --- | --- | --- | --- | --- | --- | --- | --- | --- |
| (A) | 〇 | 〇 | 〇 | 〇 | 〇 | 〇 | 〇 | (B) |
| Screening test performance |  |  |  |  |  |  |  | Availability of dried blood spots |
| (A) | 〇 | 〇 | 〇 | 〇 | 〇 | 〇 | 〇 | (B) |
| Screening test performance |  |  |  |  |  |  |  | Number of samples that can be processed |
| (A) | 〇 | 〇 | 〇 | 〇 | 〇 | 〇 | 〇 | (B) |
| Screening test performance |  |  |  |  |  |  |  | Time to obtain screening test results |
| (A) | 〇 | 〇 | 〇 | 〇 | 〇 | 〇 | 〇 | (B) |
| Screening test performance |  |  |  |  |  |  |  | Cost of screening test |
| (A) | 〇 | 〇 | 〇 | 〇 | 〇 | 〇 | 〇 | (B) |
| Screening test performance |  |  |  |  |  |  |  | Number of disease/conditions testable at once |
| (A) | 〇 | 〇 | 〇 | 〇 | 〇 | 〇 | 〇 | (B) |
| Availability of dried blood spots |  |  |  |  |  |  |  | Number of samples that can be processed |
| (A) | 〇 | 〇 | 〇 | 〇 | 〇 | 〇 | 〇 | (B) |
| Availability of dried blood spots |  |  |  |  |  |  |  | Time to obtain screening test results |
| (A) | 〇 | 〇 | 〇 | 〇 | 〇 | 〇 | 〇 | (B) |
| Availability of dried blood spots |  |  |  |  |  |  |  | Cost of screening test |
| (A) | 〇 | 〇 | 〇 | 〇 | 〇 | 〇 | 〇 | (B) |
| Availability of dried blood spots |  |  |  |  |  |  |  | Number of disease/conditions testable at once |
| (A) | 〇 | 〇 | 〇 | 〇 | 〇 | 〇 | 〇 | (B) |
| Number of samples that can be processed |  |  |  |  |  |  |  | Time to obtain screening test results |
| (A) | 〇 | 〇 | 〇 | 〇 | 〇 | 〇 | 〇 | (B) |
| Number of samples that can be processed |  |  |  |  |  |  |  | Cost of screening test |
| (A) | 〇 | 〇 | 〇 | 〇 | 〇 | 〇 | 〇 | (B) |
| Number of samples that can be processed |  |  |  |  |  |  |  | Number of disease/conditions testable at once |
| (A) | 〇 | 〇 | 〇 | 〇 | 〇 | 〇 | 〇 | (B) |
| Time to obtain screening test results |  |  |  |  |  |  |  | Cost of screening test |
| (A) | 〇 | 〇 | 〇 | 〇 | 〇 | 〇 | 〇 | (B) |
| Time to obtain screening test results |  |  |  |  |  |  |  | Number of disease/conditions testable at once |
| (A) | 〇 | 〇 | 〇 | 〇 | 〇 | 〇 | 〇 | (B) |
| Cost of screening test |  |  |  |  |  |  |  | Number of disease/conditions testable at once |

**List of Intervention items**

| 1. | Availability of clinical guidelines: There should be established, evidence-based, agreed clinical guidelines covering cut-off points for testing, additional testing and diagnosis for subjects with positive screening tests, policies for individuals to whom interventions should be provided, and standard and effective treatment strategies. |
| --- | --- |
| 2. | Availability of medical intervention covered by national health insurance: NBS participants with positive screening tests should receive appropriate interventions within the national health insurance system. |
| 3. | Scientific evidence for benefits of early intervention: There should be scientific evidence that patients identified by screening tests can benefit from appropriate early intervention. |

|  | (A) is of very strong importance | (A) is of strong importance | (A) is of moderate importance | Equal importance | (B) is of moderate importance | (B) is of strong importance | (B) is of very strong importance |  |
| --- | --- | --- | --- | --- | --- | --- | --- | --- |
| (A) | 〇 | 〇 | 〇 | 〇 | 〇 | 〇 | 〇 | (B) |
| Availability of clinical guidelines |  |  |  |  |  |  |  | Availability of medical intervention covered by national health insurance |
| (A) | 〇 | 〇 | 〇 | 〇 | 〇 | 〇 | 〇 | (B) |
| Availability of clinical guidelines |  |  |  |  |  |  |  | Scientific evidence for benefits of early intervention |
| (A) | 〇 | 〇 | 〇 | 〇 | 〇 | 〇 | 〇 | (B) |
| Availability of medical intervention covered by national health insurance |  |  |  |  |  |  |  | Scientific evidence for benefits of early intervention |

**List of Follow-up setting items**

| 1. | Post-screening follow-up system: After the patient has been diagnosed, there should be a core hospital that has a specialist with the disease/condition within the accessible range. A system to coordinate cooperation between the local hospital that the patient visits routinely or on an emergency basis and the hospital with a specialist should be well-established. |
| --- | --- |
| 2. | Availability of post-screening consultation: A system that can sufficiently explain the disease to the patients and family members who tested positive and the patient’s family (e.g., genetic counseling, brochures to explain the disease, and contacts for inquiries) should be well-established. Furthermore, this system should be standardized nationwide to allow providing information fairly to all individuals identified through screening. |

|  | (A) is of very strong importance | (A) is of strong importance | (A) is of moderate importance | Equal importance | (B) is of moderate importance | (B) is of strong importance | (B) is of very strong importance |  |
| --- | --- | --- | --- | --- | --- | --- | --- | --- |
| (A) | 〇 | 〇 | 〇 | 〇 | 〇 | 〇 | 〇 | (B) |
| Post-screening follow-up system |  |  |  |  |  |  |  | Availability of post-screening consultation |

1. Evaluation of criteria

**List of Incidence of the disease/condition items**

| 1. | Incidence of the disease/condition is ≥1/20,000. |
| --- | --- |
| 2. | Incidence of the disease/condition is ≥1/50,000 but <1/20,000. |
| 3. | Incidence of the disease/condition is ≥1/100,000 but <1/50,000. |
| 4. | Incidence of the disease/condition is ≥1/200,000 but <1/100,000. |
| 5. | Incidence of the disease/condition is <1/200,000. |

|  | (A) is of very strong importance | (A) is of strong importance | (A) is of moderate importance | Equal importance | (B) is of moderate importance | (B) is of strong importance | (B) is of very strong importance |  |
| --- | --- | --- | --- | --- | --- | --- | --- | --- |
| (A) | 〇 | 〇 | 〇 | 〇 | 〇 | 〇 | 〇 | (B) |
| ≥1/20,000 |  |  |  |  |  |  |  | ≥1/50,000 but <1/20,000 |
| (A) | 〇 | 〇 | 〇 | 〇 | 〇 | 〇 | 〇 | (B) |
| ≥1/20,000 |  |  |  |  |  |  |  | ≥1/100,000 but <1/50,000 |
| (A) | 〇 | 〇 | 〇 | 〇 | 〇 | 〇 | 〇 | (B) |
| ≥1/20,000 |  |  |  |  |  |  |  | ≥1/200,000 but <1/100,000 |
| (A) | 〇 | 〇 | 〇 | 〇 | 〇 | 〇 | 〇 | (B) |
| ≥1/20,000 |  |  |  |  |  |  |  | <1/200,000 |
| (A) | 〇 | 〇 | 〇 | 〇 | 〇 | 〇 | 〇 | (B) |
| ≥1/50,000 but <1/20,000 |  |  |  |  |  |  |  | ≥1/100,000 but <1/50,000 |
| (A) | 〇 | 〇 | 〇 | 〇 | 〇 | 〇 | 〇 | (B) |
| ≥1/50,000 but <1/20,000 |  |  |  |  |  |  |  | ≥1/200,000 but <1/100,000 |
| (A) | 〇 | 〇 | 〇 | 〇 | 〇 | 〇 | 〇 | (B) |
| ≥1/50,000 but <1/20,000 |  |  |  |  |  |  |  | <1/200,000 |
| (A) | 〇 | 〇 | 〇 | 〇 | 〇 | 〇 | 〇 | (B) |
| ≥1/100,000 but <1/50,000 |  |  |  |  |  |  |  | ≥1/200,000 but <1/100,000 |
| (A) | 〇 | 〇 | 〇 | 〇 | 〇 | 〇 | 〇 | (B) |
| ≥1/100,000 but <1/50,000 |  |  |  |  |  |  |  | <1/200,000 |
| (B) | 〇 | 〇 | 〇 | 〇 | 〇 | 〇 | 〇 | (B) |
| ≥1/200,000 but <1/100,000 |  |  |  |  |  |  |  | <1/200,000 |

**List of Onset of serious symptoms within 96 h of birth items**

| 1. | No onset of serious symptoms within 96 h of birth |
| --- | --- |
| 2. | ≥1% but <30% onset of serious symptoms within 96 h of birth |
| 3. | ≥30% but <70% onset of serious symptoms within 96 h of birth |
| 4. | ≥70% or unknown onset of serious symptoms within 96 h of birth |

|  | (A) is of very strong importance | (A) is of strong importance | (A) is of moderate importance | Equal importance | (B) is of moderate importance | (B) is of strong importance | (B) is of very strong importance |  |
| --- | --- | --- | --- | --- | --- | --- | --- | --- |
| (A) | 〇 | 〇 | 〇 | 〇 | 〇 | 〇 | 〇 | (B) |
| No onset |  |  |  |  |  |  |  | ≥1% but <30% |
| (A) | 〇 | 〇 | 〇 | 〇 | 〇 | 〇 | 〇 | (B) |
| No onset |  |  |  |  |  |  |  | ≥30% but <70% |
| (A) | 〇 | 〇 | 〇 | 〇 | 〇 | 〇 | 〇 | (B) |
| No onset |  |  |  |  |  |  |  | ≥70% or unknown |
| (A) | 〇 | 〇 | 〇 | 〇 | 〇 | 〇 | 〇 | (B) |
| ≥1% but <30% |  |  |  |  |  |  |  | ≥30% but <70% |
| (A) | 〇 | 〇 | 〇 | 〇 | 〇 | 〇 | 〇 | (B) |
| ≥1% but <30% |  |  |  |  |  |  |  | ≥70% or unknown |
| (A) | 〇 | 〇 | 〇 | 〇 | 〇 | 〇 | 〇 | (B) |
| ≥30% but <70% |  |  |  |  |  |  |  | ≥70% or unknown |

**List of Natural history of the disease/condition items**

| 1. | Natural history of the disease/condition is clear. |
| --- | --- |
| 2. | Natural history of the disease/condition is unclear. |

|  | (A) is of very strong importance | (A) is of strong importance | (A) is of moderate importance | Equal importance | (B) is of moderate importance | (B) is of strong importance | (B) is of very strong importance |  |
| --- | --- | --- | --- | --- | --- | --- | --- | --- |
| (A) | 〇 | 〇 | 〇 | 〇 | 〇 | 〇 | 〇 | (B) |
| Clear |  |  |  |  |  |  |  | Unclear |

**List of Disease burden without treatment items**

| 1. | The disease/condition has a high disease burden without treatment. |
| --- | --- |
| 2. | The disease/condition has a moderate disease burden without treatment. |
| 3. | The disease/condition has a low disease burden without treatment. |

|  | (A) is of very strong importance | (A) is of strong importance | (A) is of moderate importance | Equal importance | (B) is of moderate importance | (B) is of strong importance | (B) is of very strong importance |  |
| --- | --- | --- | --- | --- | --- | --- | --- | --- |
| (A) | 〇 | 〇 | 〇 | 〇 | 〇 | 〇 | 〇 | (B) |
| High disease burden |  |  |  |  |  |  |  | Moderate disease burden |
| (A) | 〇 | 〇 | 〇 | 〇 | 〇 | 〇 | 〇 | (B) |
| High disease burden |  |  |  |  |  |  |  | Low disease burden |
| (A) | 〇 | 〇 | 〇 | 〇 | 〇 | 〇 | 〇 | (B) |
| Moderate disease burden |  |  |  |  |  |  |  | Low disease burden |

**List of Screening test performance items**

| 1. | Screening test has high sensitivity and specificity. |
| --- | --- |
| 2. | Screening test has high sensitivity but low specificity. |
| 3. | The others |

|  | (A) is of very strong importance | (A) is of strong importance | (A) is of moderate importance | Equal importance | (B) is of moderate importance | (B) is of strong importance | (B) is of very strong importance |  |
| --- | --- | --- | --- | --- | --- | --- | --- | --- |
| (A) | 〇 | 〇 | 〇 | 〇 | 〇 | 〇 | 〇 | (B) |
| High sensitivity and specificity |  |  |  |  |  |  |  | High sensitivity but low specificity |
| (A) | 〇 | 〇 | 〇 | 〇 | 〇 | 〇 | 〇 | (B) |
| High sensitivity and specificity |  |  |  |  |  |  |  | The others |
| (A) | 〇 | 〇 | 〇 | 〇 | 〇 | 〇 | 〇 | (B) |
| High sensitivity but low specificity |  |  |  |  |  |  |  | The others |

**List of Availability of dried blood specimens items**

| 1. | Availability of dried blood specimens: yes |
| --- | --- |
| 2. | Availability of dried blood specimens: no |

|  | (A) is of very strong importance | (A) is of strong importance | (A) is of moderate importance | Equal importance | (B) is of moderate importance | (B) is of strong importance | (B) is of very strong importance |  |
| --- | --- | --- | --- | --- | --- | --- | --- | --- |
| (A) | 〇 | 〇 | 〇 | 〇 | 〇 | 〇 | 〇 | (B) |
| Availability of dried blood specimens: yes |  |  |  |  |  |  |  | Availability of dried blood specimens: no |

**List of Number of samples that can be processed items**

| 1. | ≥200 samples/day/full-time equivalent (FTE) of samples can be processed. |
| --- | --- |
| 2. | ≥100 but <200 samples/day/FTE of samples can be processed. |
| 3. | The others |

|  | (A) is of very strong importance | (A) is of strong importance | (A) is of moderate importance | Equal importance | (B) is of moderate importance | (B) is of strong importance | (B) is of very strong importance |  |
| --- | --- | --- | --- | --- | --- | --- | --- | --- |
| (A) | 〇 | 〇 | 〇 | 〇 | 〇 | 〇 | 〇 | (B) |
| ≥200 samples/day/FTE |  |  |  |  |  |  |  | ≥100 but <200 samples/day/FTE |
| (A) | 〇 | 〇 | 〇 | 〇 | 〇 | 〇 | 〇 | (B) |
| ≥200 samples/day/FTE |  |  |  |  |  |  |  | The others |
| (A) | 〇 | 〇 | 〇 | 〇 | 〇 | 〇 | 〇 | (B) |
| ≥100 but <200 samples/day/FTE |  |  |  |  |  |  |  | The others |

**List of Time to obtain screening test results items**

| 1. | Time to obtain screening test results is <1 day. |
| --- | --- |
| 2. | Time to obtain screening test results is ≥1 but <2 days. |
| 3. | Time to obtain screening test results is ≥2 days. |

|  | (A) is of very strong importance | (A) is of strong importance | (A) is of moderate importance | Equal importance | (B) is of moderate importance | (B) is of strong importance | (B) is of very strong importance |  |
| --- | --- | --- | --- | --- | --- | --- | --- | --- |
| (A) | 〇 | 〇 | 〇 | 〇 | 〇 | 〇 | 〇 | (B) |
| <1 day |  |  |  |  |  |  |  | ≥1 but <2 days |
| (A) | 〇 | 〇 | 〇 | 〇 | 〇 | 〇 | 〇 | (B) |
| <1 day |  |  |  |  |  |  |  | ≥2 days |
| (A) | 〇 | 〇 | 〇 | 〇 | 〇 | 〇 | 〇 | (B) |
| ≥1 but <2 days |  |  |  |  |  |  |  | ≥2 days |

**List of Cost of screening test items**

| 1. | Cost of screening test is <500 Japanese yen (JPY). |
| --- | --- |
| 2. | Cost of screening test is 500–999 JPY. |
| 3. | Cost of screening test is 1000–4999 JPY. |
| 4. | Cost of screening test is ≥5000 JPY. |

|  | (A) is of very strong importance | (A) is of strong importance | (A) is of moderate importance | Equal importance | (B) is of moderate importance | (B) is of strong importance | (B) is of very strong importance |  |
| --- | --- | --- | --- | --- | --- | --- | --- | --- |
| (A) | 〇 | 〇 | 〇 | 〇 | 〇 | 〇 | 〇 | (B) |
| <500 Japanese yen (JPY) |  |  |  |  |  |  |  | 500–999 JPY |
| (A) | 〇 | 〇 | 〇 | 〇 | 〇 | 〇 | 〇 | (B) |
| <500 Japanese yen (JPY) |  |  |  |  |  |  |  | 1000–4999 JPY |
| (A) | 〇 | 〇 | 〇 | 〇 | 〇 | 〇 | 〇 | (B) |
| <500 Japanese yen (JPY) |  |  |  |  |  |  |  | ≥5000 JPY |
| (A) | 〇 | 〇 | 〇 | 〇 | 〇 | 〇 | 〇 | (B) |
| 500–999 JPY |  |  |  |  |  |  |  | 1000–4999 JPY |
| (A) | 〇 | 〇 | 〇 | 〇 | 〇 | 〇 | 〇 | (B) |
| 500–999 JPY |  |  |  |  |  |  |  | ≥5000 JPY |
| (A) | 〇 | 〇 | 〇 | 〇 | 〇 | 〇 | 〇 | (B) |
| 1000–4999 JPY |  |  |  |  |  |  |  | ≥5000 JPY |

**List of Number of diseases/conditions testable at once items**

| 1. | ≥4 diseases/conditions testable at once |
| --- | --- |
| 2. | 2–3 diseases/conditions testable at once |
| 3. | 1 diseases/conditions testable at once |

|  | (A) is of very strong importance | (A) is of strong importance | (A) is of moderate importance | Equal importance | (B) is of moderate importance | (B) is of strong importance | (B) is of very strong importance |  |
| --- | --- | --- | --- | --- | --- | --- | --- | --- |
| (A) | 〇 | 〇 | 〇 | 〇 | 〇 | 〇 | 〇 | (B) |
| ≥4 |  |  |  |  |  |  |  | 2–3 |
| (A) | 〇 | 〇 | 〇 | 〇 | 〇 | 〇 | 〇 | (B) |
| ≥4 |  |  |  |  |  |  |  | 1 |
| (A) | 〇 | 〇 | 〇 | 〇 | 〇 | 〇 | 〇 | (B) |
| 2–3 |  |  |  |  |  |  |  | 1 |

**List of Availability of clinical guidelines items**

| 1. | Clinical guidelines are available. |
| --- | --- |
| 2. | Clinical guidelines are partially available. |
| 3. | Clinical guidelines are not available. |

|  | (A) is of very strong importance | (A) is of strong importance | (A) is of moderate importance | Equal importance | (B) is of moderate importance | (B) is of strong importance | (B) is of very strong importance |  |
| --- | --- | --- | --- | --- | --- | --- | --- | --- |
| (A) | 〇 | 〇 | 〇 | 〇 | 〇 | 〇 | 〇 | (B) |
| Available |  |  |  |  |  |  |  | Partially available |
| (A) | 〇 | 〇 | 〇 | 〇 | 〇 | 〇 | 〇 | (B) |
| Available |  |  |  |  |  |  |  | Not available |
| (A) | 〇 | 〇 | 〇 | 〇 | 〇 | 〇 | 〇 | (B) |
| Partially available |  |  |  |  |  |  |  | Not available |

**List of Availability of medical interventions covered by national health insurance items**

| 1. | Medical interventions covered by national health insurance is available. |
| --- | --- |
| 2. | Medical interventions covered by national health insurance is partially available. |
| 3. | The others |

|  | (A) is of very strong importance | (A) is of strong importance | (A) is of moderate importance | Equal importance | (B) is of moderate importance | (B) is of strong importance | (B) is of very strong importance |  |
| --- | --- | --- | --- | --- | --- | --- | --- | --- |
| (A) | 〇 | 〇 | 〇 | 〇 | 〇 | 〇 | 〇 | (B) |
| Available |  |  |  |  |  |  |  | Partially available |
| (A) | 〇 | 〇 | 〇 | 〇 | 〇 | 〇 | 〇 | (B) |
| Available |  |  |  |  |  |  |  | The others |
| (A) | 〇 | 〇 | 〇 | 〇 | 〇 | 〇 | 〇 | (B) |
| Partially available |  |  |  |  |  |  |  | The others |

**List of Scientific evidence of the benefits of early intervention items**

| 1. | Scientific evidence of the benefits of early intervention: yes |
| --- | --- |
| 2. | Scientific evidence of the benefits of early intervention: some |
| 3. | Scientific evidence of the benefits of early intervention: no |

|  | (A) is of very strong importance | (A) is of strong importance | (A) is of moderate importance | Equal importance | (B) is of moderate importance | (B) is of strong importance | (B) is of very strong importance |  |
| --- | --- | --- | --- | --- | --- | --- | --- | --- |
| (A) | 〇 | 〇 | 〇 | 〇 | 〇 | 〇 | 〇 | (B) |
| Yes |  |  |  |  |  |  |  | Some |
| (A) | 〇 | 〇 | 〇 | 〇 | 〇 | 〇 | 〇 | (B) |
| Yes |  |  |  |  |  |  |  | No |
| (A) | 〇 | 〇 | 〇 | 〇 | 〇 | 〇 | 〇 | (B) |
| Some |  |  |  |  |  |  |  | No |

**List of Post-screening follow-up system items**

| 1. | Post-screening follow-up system is well-established. |
| --- | --- |
| 2. | Post-screening follow-up system is partially established. |
| 3. | Post-screening follow-up system is not established. |

|  | (A) is of very strong importance | (A) is of strong importance | (A) is of moderate importance | Equal importance | (B) is of moderate importance | (B) is of strong importance | (B) is of very strong importance |  |
| --- | --- | --- | --- | --- | --- | --- | --- | --- |
| (A) | 〇 | 〇 | 〇 | 〇 | 〇 | 〇 | 〇 | (B) |
| Well-established |  |  |  |  |  |  |  | Partially established |
| (A) | 〇 | 〇 | 〇 | 〇 | 〇 | 〇 | 〇 | (B) |
| Well-established |  |  |  |  |  |  |  | Not established |
| (A) | 〇 | 〇 | 〇 | 〇 | 〇 | 〇 | 〇 | (B) |
| Partially established |  |  |  |  |  |  |  | Not established |

**List of Availability of post-screening consultation items**

| 1. | Post-screening consultation is available. |
| --- | --- |
| 2. | Post-screening consultation is partially available. |
| 3. | Post-screening consultation is not available. |

|  | (A) is of very strong importance | (A) is of strong importance | (A) is of moderate importance | Equal importance | (B) is of moderate importance | (B) is of strong importance | (B) is of very strong importance |  |
| --- | --- | --- | --- | --- | --- | --- | --- | --- |
| (A) | 〇 | 〇 | 〇 | 〇 | 〇 | 〇 | 〇 | (B) |
| Available |  |  |  |  |  |  |  | Partially available |
| (A) | 〇 | 〇 | 〇 | 〇 | 〇 | 〇 | 〇 | (B) |
| Available |  |  |  |  |  |  |  | Not available |
| (A) | 〇 | 〇 | 〇 | 〇 | 〇 | 〇 | 〇 | (B) |
| Partially available |  |  |  |  |  |  |  | Not available |

**List of Economic evaluation items**

| 1. | Clear scientific evidence is available. |
| --- | --- |
| 2. | Some scientific evidence is available. |
| 3. | The others |

|  | (A) is of very strong importance | (A) is of strong importance | (A) is of moderate importance | Equal importance | (B) is of moderate importance | (B) is of strong importance | (B) is of very strong importance |  |
| --- | --- | --- | --- | --- | --- | --- | --- | --- |
| (A) | 〇 | 〇 | 〇 | 〇 | 〇 | 〇 | 〇 | (B) |
| Scientific evidence is available |  |  |  |  |  |  |  | Some scientific evidence is available |
| (A) | 〇 | 〇 | 〇 | 〇 | 〇 | 〇 | 〇 | (B) |
| Scientific evidence is available |  |  |  |  |  |  |  | The others |
| (A) | 〇 | 〇 | 〇 | 〇 | 〇 | 〇 | 〇 | (B) |
| Some scientific evidence is available |  |  |  |  |  |  |  | The others |

This questionnaire was conducted in Japanese.
